# Immune checkpoint inhibitor-induced hemophagocytic lymphohistiocytosis in lung cancer: a case series

**DOI:** 10.37349/etat.2025.1002347

**Published:** 2025-11-12

**Authors:** Abdul Wali Khan, Simran Chandra, Himil Mahadevia, Janakiraman Subramanian, Ben Ponvilawan, Dhruv Bansal

**Affiliations:** IRCCS Istituto Romagnolo per lo Studio dei Tumori (IRST) “Dino Amadori”, Italy; ^1^Department of Internal Medicine, University of Missouri–Kansas City School of Medicine, Kansas City, MO 64108, USA; ^2^Inova Schar Cancer Institute, Fairfax, VA 22031, USA; ^3^Evanston Hospital, Endeavor Health, Evanston, IL 60201, USA

**Keywords:** immune checkpoint inhibitors, HLH, lung cancer

## Abstract

Immune checkpoint inhibitors (ICIs) are established treatments for various malignancies, including lung, kidney, and colorectal cancers. However, their broad use has led to an increase in immune-related adverse events (irAEs), with thyroiditis, colitis, and pneumonitis being the most common. Hemophagocytic lymphohistiocytosis (HLH) is a rare but severe irAE characterized by excessive immune activation, leading to systemic inflammation and multi-organ dysfunction. We present three cases of ICI-induced HLH in patients with lung cancer who were treated with ICIs. All patients showed elevated inflammatory markers and responded to high-dose corticosteroids without the addition of etoposide. These cases underscore the importance of early recognition and treatment of HLH in patients receiving ICIs to mitigate morbidity and mortality. Large-scale studies are needed to establish standardized guidelines for diagnosing and managing ICI-induced HLH.

## Introduction

Immune checkpoint inhibitors (ICIs) are one of the main treatments for various malignancies, including lung, kidney, and colorectal cancers [1–3]. As their indications broaden, there has been a significant rise in the global reporting of immune-related adverse events (irAEs). These irAEs can affect any organ system, with thyroiditis, colitis, and pneumonitis being the most reported serious conditions [4].

Hemophagocytic lymphohistiocytosis (HLH) is a rare but aggressive and life-threatening syndrome caused by immune hyperactivation, with common triggers including viral infections, malignancies, and rheumatologic disorders. The pathophysiology of HLH involves the disruption of normal immunoregulatory pathways, leading to excessive and persistent activation of histiocytes, NK cells, and CD8^+^ cytotoxic lymphocytes, resulting in systemic immune dysregulation, which can manifest as persistent high fevers, pancytopenia, hepatitis, coagulopathy, kidney dysfunction, respiratory deterioration, and, in some cases, neurologic symptoms [5].

Given that HLH is a life-threatening condition, prompt initiation of treatment is essential. However, delays in diagnosis and challenges in obtaining a definitive diagnosis through advanced testing significantly hinder timely intervention. Treatment typically involves corticosteroids and immunosuppressive therapies, with the HLH-94 protocol—utilizing dexamethasone and etoposide—as the most common regimen [6].

Despite several case reports suggesting a potential link between ICI and the development of HLH, data on ICI-related HLH remains scarce [7–10]. Here, we present three cases of ICI-related HLH.

## Case presentations

### Case 1

A 72-year-old female with relapsed small-cell lung cancer of the left lung with metastases to paratracheal and hilar lymph nodes and recurrence in the right lung presented to the oncology clinic with complaints of fatigue, subjective fever, and nausea 19 days after receiving the first cycle of carboplatin, etoposide, and atezolizumab for recurrent disease. The physical exam did not reveal hepatosplenomegaly. She previously received four cycles of concurrent chemoradiation with cisplatin and etoposide eighteen months before this presentation. Initial laboratory workup showed markedly elevated inflammatory markers and elevated liver enzymes, prompting admission for further management. The laboratory results of pre-ICI treatment and on the day of admission are compared in Table 1.

**Table 1:** Laboratory results of a patient with recurrent small-cell lung cancer and HLH.

**Laboratory test**	**Day of admission**	**Before receiving atezolizumab**	**Reference range**
ESR	69 mm/h	Not available	0–20 mm/h
CRP	330 mg/L	Not available	0–10 mg/L
WBC count	4,800 Th/uL	4,300 Th/uL	4,000–11,000 Th/uL
Hemoglobin	11.5 g/dL	13.0 g/dL	12–15 g/dL
Platelets	179,000 Th/uL	189,000 Th/uL	140,000–400,000 Th/uL
Aspartate aminotransferase	239 U/L	30 U/L	0–34 U/L
Alanine aminotransferase	159 U/L	26 U/L	0–34 U/L
Alkaline phosphatase	430 U/L	136 U/L	44–150 U/L
Total bilirubin	0.5 mg/dL	0.3 mg/dL	0.2–1.1 mg/dL

CRP: C-reactive peptide; ESR: erythrocyte sedimentation rate; HLH: hemophagocytic lymphohistiocytosis; Th: thousand; WBC: white blood cell.

Further workup revealed elevated ferritin level of 2,049 ng/mL (normal: 7.3–270 ng/mL), triglyceride level of 187 mg/dL (normal: < 150 mg/dL), fibrinogen level of 786 mg/dL (normal: 146–390 mg/dL), D-dimer of 4.36 mg/mL fibrinogen equivalent unit (FEU) (normal: 0.0–0.4 mg/mL FEU), positive ANA titers and creatine kinase level of 157 U/L (normal: 34–145 U/L). Of note, her soluble interleukin-2 receptor (sIL-2R) level was elevated at 750 U/mL (normal: 223–710 U/mL). Her clinical presentation, along with elevated ferritin, IL-2R, and H-score of 105, was highly suggestive of atezolizumab-induced HLH. Of note, the bone marrow biopsy and flow cytometry were unremarkable. Atezolizumab was discontinued, while carboplatin and etoposide were continued. In addition, she was started on a dexamethasone taper per the HLH-94 protocol. She did not receive etoposide as typically administered per the HLH-94 protocol. Her clinical symptoms were significantly improved in two weeks, and she is currently following up in the clinic for the management of her recurrent small-cell lung cancer.

### Case 2

A 77-year-old female non-smoker with *BRAF V600E*-positive stage IV right lower lobe lung adenocarcinoma with thoracic lymph node, intramuscular, and intracranial metastases presented to the oncology clinic with complaints of fever (101°F), fatigue, significant weakness, nausea, vomiting, and worsening shortness of breath 25 days after completing the first cycle of pembrolizumab. The physical exam did not show any hepatosplenomegaly. She was subsequently admitted to the hospital for further management. On admission, laboratory workup markedly elevated ferritin of more than 16,500 ng/mL (normal: 7.3–270 ng/mL), sIL-2R of 2,500 U/mL (normal: 223–710 U/mL), and triglycerides of 243 mg/dL (normal: < 150 mg/dL). Fibrinogen level was low at 127 mg/dL (normal: 146–390 mg/dL). Additionally, she was found to have pancytopenia and elevated lactate dehydrogenase (LDH) at 483 U/L (normal: 120–246 U/L). A comparison of lab work before receiving the pembrolizumab and on admission is mentioned in Table 2. Abdominal ultrasound was negative for hepatosplenomegaly.

**Table 2:** Laboratory results of a patient with metastatic lung adenocarcinoma and HLH.

**Laboratory test**	**Day of admission**	**Before receiving pembrolizumab**	**Reference range**
ESR	Not available	Not available	0–20 mm/h
CRP	192 mg/L	Not available	0–10 mg/L
WBC count	3,070 Th/uL	5200 Th/uL	4,000–11,000 Th/uL
Hemoglobin	7.5 g/dL	10.5 g/dL	12–15 g/dL
Platelets	15,000 Th/uL	210 Th/uL	140,000–400,000 Th/uL
Aspartate aminotransferase	46 U/L	34 U/L	0–34 U/L
Alanine aminotransferase	64 U/L	28 U/L	0–34 U/L
Alkaline phosphatase	136 U/L	122 U/L	44–150 U/L
Total bilirubin	0.2 mg/dL	0.3 mg/dL	0.2–1.1 mg/dL

CRP: C-reactive peptide; ESR: erythrocyte sedimentation rate; HLH: hemophagocytic lymphohistiocytosis; Th: thousand; WBC: white blood cell.

An extensive infectious workup, including polymerase chain reaction (PCR) of *Bartonella* spp., parvovirus B19 virus, herpes simplex virus (HSV)-1, HSV-2, cytomegalovirus (CMV), human herpesvirus (HHV)-6, and fungal blood culture, was all negative. A bone marrow biopsy was also performed, which was unremarkable. Her H-score was 210, which raised a strong suspicion of pembrolizumab-induced HLH. Subsequently, pembrolizumab was discontinued, methylprednisolone 125 mg was administered intravenously for five days while inpatient, and then subsequently discharged on oral dexamethasone 10 mg/m^2^ followed by taper over two months as outlined in the HLH-94 protocol. Her sIL-2R level, ferritin, and pancytopenia normalized three months later, and fatigue resolved.

A follow-up positron-emission tomography (PET) scan and brain magnetic resonance imaging (MRI) revealed intracranial cancer progression but significant regression of extracranial disease burden from cancer. For brain metastasis, she underwent stereotactic radiosurgery and remained off systemic treatment for five months. Subsequently, she experienced a relapse of lung adenocarcinoma, manifesting as lung nodules, thoracic adenopathy, peritoneal carcinomatosis, and intramuscular metastases. Pembrolizumab was initiated, and two months later, due to rising ferritin, progressive fatigue, and concern for recurrence of HLH, she was transitioned to dabrafenib and trametinib. An extensive workup to rule out other etiologies for fatigue, such as endocrinopathies, was performed and did not reveal any obvious anomalies. A CT chest, abdomen, and pelvis was performed three months later and revealed progressive disease. She decided to pursue hospice care, and dabrafenib and trametinib were discontinued.

### Case 3

A 52-year-old female with a history of endometrial cancer for which she underwent total hysterectomy with bilateral salpingo-oophorectomy, followed by six cycles of adjuvant pembrolizumab, paclitaxel, and carboplatin. Four months after the last dose of pembrolizumab, she presented to the clinic with complaints of fatigue, fever, and night sweats. Her physical exam was unrevealing for lymphadenopathy or hepatosplenomegaly. Labs revealed significantly elevated ferritin of 8,491 ng/mL (normal: 7.3–270 ng/mL), C-reactive peptide (CRP) of 52 mg/L, low fibrinogen of 137 mg/dL (normal: 146–390 mg/dL), and pancytopenia with white blood cell (WBC) count of 2,100 Th/uL , hemoglobin of 6.3 g/dL, and platelets of 16,000 Th/uL. Notably, the sIL-2R level was markedly elevated at 3,338 U/mL. However, the triglyceride and liver enzyme levels were within normal limits. A bone marrow biopsy was done to evaluate further the pancytopenia, which revealed no significant anomalies forty percent cellularity, which is normal for age.

Based on elevated ferritin and sIL-2R levels and an elevated H-score of 209, the patient was diagnosed with pembrolizumab-induced HLH. She was subsequently started on dexamethasone per HLH-94 protocol, which significantly improved her symptoms within one week after treatment. Repeat lab work also showed improved pancytopenia, ferritin, and sIL-2R levels.

## Discussion

A growing body of literature reports cases of HLH associated with ICI therapy [7–10]. Tumor cells employ multiple strategies to avoid detection and destruction by the immune system, one of which involves exploiting immune checkpoint pathways that suppress immune responses. ICIs are designed to disrupt these suppressive mechanisms, thereby boosting the immune system’s ability to target and destroy cancer cells. By inhibiting critical immune regulators—such as CTLA-4, PD-1, and PD-L1—ICIs unleash a more potent antitumor immune response. However, this enhanced immune activation comes with risks. In rare instances, the removal of these inhibitory brakes can lead to an overactive immune state, potentially resulting in severe inflammatory conditions [11]. One such condition is HLH, a rare but life-threatening syndrome thought to be driven by excessive activation of cytotoxic T cells and macrophages, along with heightened interferon-γ signaling [5].

Although HLH has been reported with various classes of ICIs, including PD-1, PD-L1, and, less commonly, CTLA-4 inhibitors, the current literature is insufficient to determine whether one class carries a higher risk. In our series, HLH occurred following treatment with both PD-1 (pembrolizumab) and PD-L1 (atezolizumab) inhibitors, suggesting that the risk may not be restricted to a specific agent but rather reflect a class-wide immune activation mechanism.

Early diagnosis of ICI-induced HLH remains a significant clinical challenge due to its nonspecific presentation and overlap with other common conditions, such as sepsis or cancer progression. Prompt recognition is critical, as delays in treatment contribute to increased morbidity and mortality. Given these challenges, there is growing interest in identifying reliable biomarkers and clinical predictors to facilitate earlier diagnosis.

**Figure 1:**
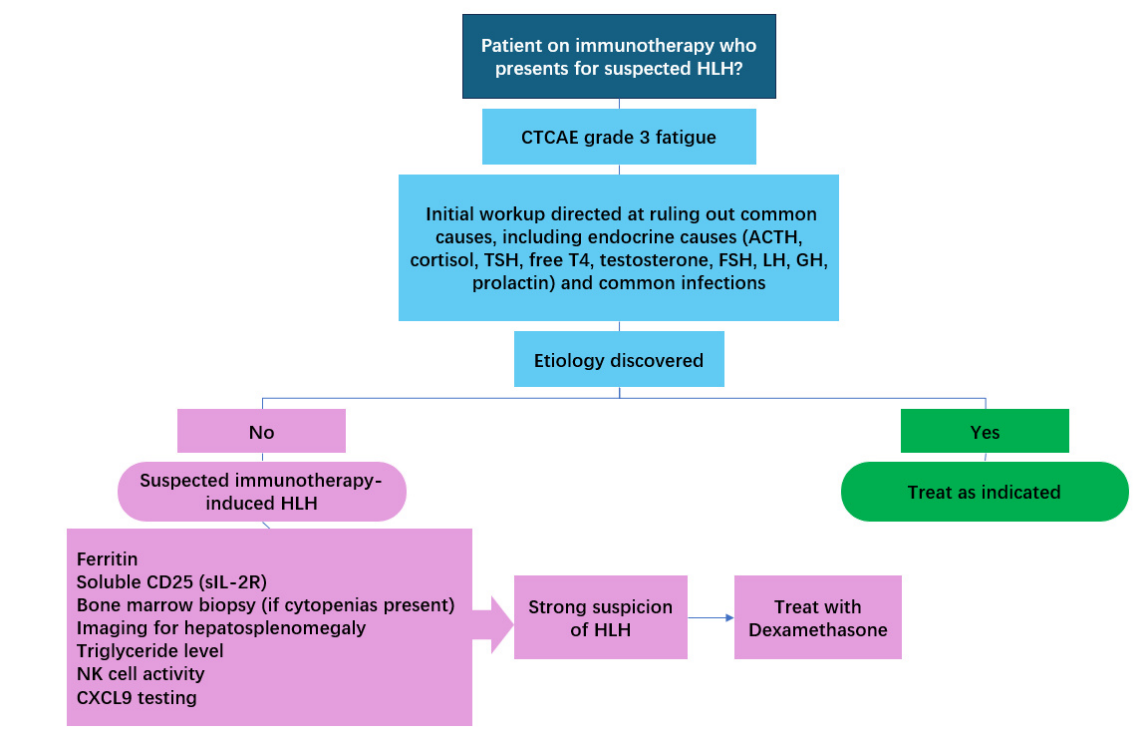
**Approach to diagnosis and treatment of immunotherapy-induced hemophagocytic lymphohistiocytosis (HLH).** CTCAE: common terminology criteria for adverse events; CXCL9: chemokine (C-X-C motif) ligand 9; GH: growth hormone; sIL-2R: soluble interleukin-2 receptor.

The HLH-2004 protocol provides a framework for diagnosing HLH, outlining clinical, laboratory, and pathological criteria, including fever, splenomegaly, cytopenia, hypertriglyceridemia, hypofibrinogenemia, hemophagocytosis in hematopoietic or lymphoid organs, low or absent NK cell activity, hyperferritinemia, elevated soluble CD25, and elevated chemokine (C-X-C motif) ligand 9 (CXCL9) [12]. Although the protocol recommends meeting at least five out of the nine criteria for diagnosis, the high mortality associated with untreated HLH often necessitates initiating treatment based on strong clinical suspicion, even if not all criteria are fulfilled (Figure 1). In practice, this approach is particularly relevant when other differential diagnoses have been excluded and the clinical picture is highly suggestive of HLH. For example, in Case 1, treatment was initiated despite not fully meeting the HLH-2004 criteria, given the overall presentation and the life-threatening consequences of delayed therapy.

Another diagnostic tool for HLH is the H-score. In addition to the previously mentioned diagnostic criteria, it incorporates factors such as known immunosuppression, hepatomegaly, and elevated AST levels. In the original study that established the H-score, patients diagnosed with reactive hemophagocytic syndrome had a median score of 230 (IQR: 203–257), whereas those without the diagnosis had a median score of 125 (IQR: 91–150). The likelihood of having hemophagocytic syndrome ranges from less than 1% for H-scores of ≤ 90 to over 99% for H-scores of ≥ 250 [13].

Among laboratory markers, ferritin and sIL-2R are particularly valuable, showing marked elevation in HLH and featuring in both HLH-2004 and H-score criteria. There is evidence to suggest that ferritin and sIL-2R levels correlate with disease activity [5]. Exploratory studies suggest that cytokines such as CXCL9, interferon-γ, and IL-13 might further improve the diagnostic accuracy of irAEs, though these are not yet validated for routine clinical use [14].

Treatment of HLH traditionally follows the HLH-94 protocol, combining corticosteroids and immunosuppressive agents like etoposide, alongside addressing underlying triggers [6]. However, data specifically addressing the treatment of ICI-induced HLH remain limited. Several case reports indicate that this subtype of HLH may respond effectively to high-dose corticosteroids alone [7–10]. Given that ICI-induced HLH likely represents an irAEs driven by heightened immune activation rather than a primary genetic disorder, the favorable response to steroids is not unexpected. In all three of our cases, high-dose corticosteroid therapy led to rapid and sustained clinical improvement, with resolution of symptoms within weeks and normalization of ferritin and sIL-2R levels within three months. Etoposide was omitted in these patients due to their prompt response to steroids, an approach supported by emerging evidence suggesting that cytotoxic agents may not be necessary in managing ICI-induced HLH. In Table 3, we have included the timeline of HLH presentation and steroid response in ICI therapy for our patients.

**Table 3:** Timeline of HLH presentation and steroid response in ICI therapy.

**Timeline event**	**Case 1: SCLC, atezolizumab**	**Case 2: NSCLC, pembrolizumab**	**Case 3: endometrial CA, pembrolizumab**
**Received chemo/ICI**	Day 0: carboplatin, etoposide, atezolizumab	Day 0: pembrolizumab	Day 0: pembrolizumab
**Day of onset of HLH symptoms**	Day 19: fatigue, fever, nausea	Day 25: fever, fatigue, SOB, nausea, vomiting	~120 days post-treatment: fatigue, fever, night sweats
**Day steroid started**	Day 19	Day 25	Day 120
**Final outcome**	Improved in 2 weeks; continued cancer follow-up	Initial improvement; HLH recurrence after rechallenge; transitioned to hospice	Improved within 1 week; labs normalized

ICI: immune checkpoint inhibitor; HLH: hemophagocytic lymphohistiocytosis.

Notably, there is increasing interest in the potential to reintroduce ICIs following the resolution of these events. A cohort study involving patients with non-small cell lung cancer NSCLC who experienced irAEs suggested improved overall survival with the reintroduction of immunotherapy, particularly in patients who had not responded to treatment before the onset of irAEs [15]. In Case 2, pembrolizumab was reintroduced, but with a recurrence of symptoms. Together with other reports, these findings highlight the need for cautious consideration and close monitoring when restarting ICIs after HLH.

## Conclusions

As the use of ICIs becomes more widespread, a heightened awareness of ICI-induced HLH is critical to avoid the delay in diagnosis and treatment to reduce morbidity and mortality. In our case series, all three patients responded favorably to a steroid-only regimen based on the HLH-94 protocol. This warrants larger studies in the future to investigate and verify the clinical utility and effectiveness of this regimen in this patient population.

## Abbreviations

CXCL9: chemokine (C-X-C motif) ligand 9
FEU: fibrinogen equivalent unit
HLH: hemophagocytic lymphohistiocytosis
HSV: herpes simplex virus
ICIs: immune checkpoint inhibitors
irAEs: immune-related adverse events
sIL-2R: soluble interleukin-2 receptor

## Declarations

### Availability of data and materials

The patient’s data is available through Saint Luke’s Hospital electronic medical records. More information can be obtained by contacting the corresponding author.

### Copyright

© The Author(s) 2025.
